# The Importance of Platelets Response during Antiplatelet Treatment after Ischemic Stroke—Between Benefit and Risk: A Systematic Review

**DOI:** 10.3390/ijms23031043

**Published:** 2022-01-18

**Authors:** Joanna Sikora, Aleksandra Karczmarska-Wódzka, Joanna Bugieda, Przemysław Sobczak

**Affiliations:** 1 Research and Education Unit for Experimental Biotechnology, Department of Transplantology and General Surgery, Faculty of Medicine, Collegium Medicum in Bydgoszcz, Nicolaus Copernicus University in Toruń, 85-094 Bydgoszcz, Poland; akar@cm.umk.pl (A.K.-W.); joanna.bugieda@cm.umk.pl (J.B.); 2 Department of Laboratory Medicine, Collegium Medicum in Bydgoszcz, Nicolaus Copernicus University in Toruń, 85-094 Bydgoszcz, Poland; przemyslawsobczak02@gmail.com

**Keywords:** antiplatelet drugs, ischemic stroke, monitoring of platelet function, personalized treatment

## Abstract

Ischemic stroke is a disease related to abnormal blood flow that leads to brain dysfunction. The early and late phases of the disease are distinguished. A distinction is made between the early and late stages of the disease, and the best effect in treating an ischemic stroke is usually achieved within the first hours after the onset of symptoms. This review looked at studies platelet activity monitoring studies to determine the risks and benefits of various approaches including antiplatelet therapy. A study was conducted on recently published literature based on PRISMA. This review includes 32 research articles directly addressing the importance of monitoring platelet function during antiplatelet therapy (dual or monotherapy) after ischemic stroke. In patients with transient ischemic attack or ischemic stroke, antiplatelet therapy can reduce the risk of stroke by 11–15%, assuming that patients respond well. Secondary prevention results are dependent on platelet reactivity, meaning that patients do not respond equally to antiplatelet therapy. It is very important that aspirin-resistant patients can benefit from the use of dual antiplatelet therapy. The individualized approach to secondary stroke prevention is to administer the most appropriate drug at the correct dose and apply the optimal therapeutic procedure to the individual patient.

## 1. Introduction

Ischemic stroke (IS), as defined by the World Health Organization (WHO) from 1970, is a sudden onset of ongoing focal or generalized brain dysfunction longer than 24 h (if they do not lead to death) and caused solely by cerebral vascular causes blood flow. The early phase (acute phase) of a stroke is the arbitrary period between the onset of symptoms and the end of the 7th day of the disease. After this time, there is the late phase of the disease. The best treatment for IS can be achieved during the first hours after the onset of symptoms, therefore, urgent action is required. This reason should be treated by all medical services as a condition requiring immediate action according to certain rules, similarly such as an acute myocardial infarction or multiple organ trauma. Specialized medical treatment should be started immediately, especially if the patient may be a potential candidate for reperfusion therapy (intravenous thrombolysis or thrombectomy) [1].

Well-worded post-stroke care has been shown to improve treatment outcomes. This is due to a better understanding of the disease and new potential therapies. Nevertheless, there is still a need for analyses of pathophysiological mechanisms depending on the type of stroke in order to personalize treatment [2]. Embolic stroke occurs when a blood clot that forms elsewhere is released from there and finally becomes stuck in a smaller brain vessel, causing an ischemic event. Blood clots that lead to embolic stroke can form anywhere, however, they usually come from the heart or arteries of the upper chest and neck. After a clot is released, it passes through the bloodstream to the brain. When it enters a blood vessel that is too small to allow it to pass, the clot becomes stuck in the blood vessel. This blocks blood flow to the brain and blockages are called emboli. Ischemic occlusion generates thrombotic and embolic conditions in the brain. In thrombosis, blood flow is affected by the narrowing of vessels due to atherosclerosis. The build-up of plaque will eventually constrict the vascular chamber and form clots, causing thrombotic stroke. In an embolic stroke, decreased blood flow to the brain region causes an embolism; the blood flow to the brain reduces, causing severe stress and untimely cell necrosis. Necrosis is followed by disruption of the plasma membrane, organelle swelling and leaking of cellular contents into extracellular space, and loss of neuronal function. Other key events contributing to stroke pathology are inflammation, increased intracellular calcium levels, loss of homeostasis, energy failure, acidosis, free radical-mediated toxicity, cytokine-mediated cytotoxicity, impairment of the blood–brain barrier, oxidative stress, and infiltration of leukocytes [3,4].

In the case of AIS (which means the closure of, e.g., cerebral arteries, spinal cord, or retina, resulting in a tissue infarction in this place, which in turn causes a sudden neurological deficit), antiplatelet drugs are used in primary and secondary prevention. Primary prevention aims to prevent disease or injury before it ever occurs. This is carried out by preventing exposures to hazards that cause disease or injury, altering unhealthy or unsafe behaviors that can lead to disease or injury, and increasing resistance to disease or injury should exposure occur. Primary prevention in IS includes lifestyle modification, which includes diet, weight control, physical activity and not smoking, and pharmacotherapy to control the risk factors for cardiovascular disease in each patient, such as hypertension, diabetes, and atrial fibrillation. Secondary prevention is used to prevent the recurrence of ischemic events. In order to reduce the degree of subsequent stroke episodes after the first stroke, it is essential for quick implementation of intensive and effective treatment of patients in the early stages of IS. The risk of the occurrence of subsequent stroke episodes should be reduced using effective drugs of secondary prevention. The risk after IS relapse may reach 10–12% in the first year and the cumulative risk of recurrence stroke within 5 years is as high as 30–40%. The frequency of relapses remains dependent on the subtype of stroke and is highest in patients with embolic strokes of cardiac origin and in cases of severe stenosis carotid arteries. Unfortunately, over 15% of patients may suffer a heart attack in the heart muscle and a similar percentage of patients will die because of vascular diseases [5].

Platelets are involved in the pathophysiology of IS because they take part in the mechanism of blood clot formation. Platelet adhesion, activation, and aggregation are critical events in hemostasis and thrombosis. At the site of vascular injury, platelet accumulation (i.e., adhesion and aggregation) constitutes the first wave of hemostasis. Blood coagulation, initiated by the coagulation cascades, is the second wave of thrombin generation and enhance phosphatidylserine exposure, can markedly potentiate cell-based thrombin generation, and enhance blood coagulation. Excessive activation of thrombocytes results in increased thrombin production as well as abnormal thrombus production. The accumulation of thrombin at sites of vascular injury provides one of the key ways for recruiting platelets into a growing hemostatic plug [6,7]. After IS for the prevention of further thrombotic incidents, patients are usually given either aspirin (ASA) or clopidogrel, however many patients are resistant to these drugs. There are other antiplatelet treatment strategies, including a combination of aspirin and dipyridamole, or the use of ticagrelor, but we have not identified studies that have used such antiplatelet therapy and monitored platelet activity. In this review, we will focus on two antiplatelet drugs: aspirin and clopidogrel, used in different combinations (together or separately). A clinical term often used in this situation is high on-treatment platelet reactivity (HTPR). Various types of platelet function tests are used to personalize antiplatelet therapy [8]. For the study of platelet function, the point-of-care devices are used: PFA-100^®^ (PFA) (Siemens, Malvern, PA, USA), VerifyNow^TM^ (Werfen, Bedford, MA, USA), Multiplate^®^Analyzer (Multiplate) (Roche Diagnostics, Mannheim, Germany), TEG^®^/ROTEM^®^ (TEG), which are simple to perform compared to more advanced methods such as light transmission aggregometer (LTA) and flow cytometry [9]. The advantages and disadvantages of platelet function tests are included in Table 1. Several older studies have suggested that the risks of long-term dual antiplatelet therapy for the secondary prevention of IS outweigh the potential benefits. We focused on studies where platelet activity was monitored to verify the risks and benefits of different therapeutic approaches associated with antiplatelet therapy.

## 2. Methods

**Figure 1:**
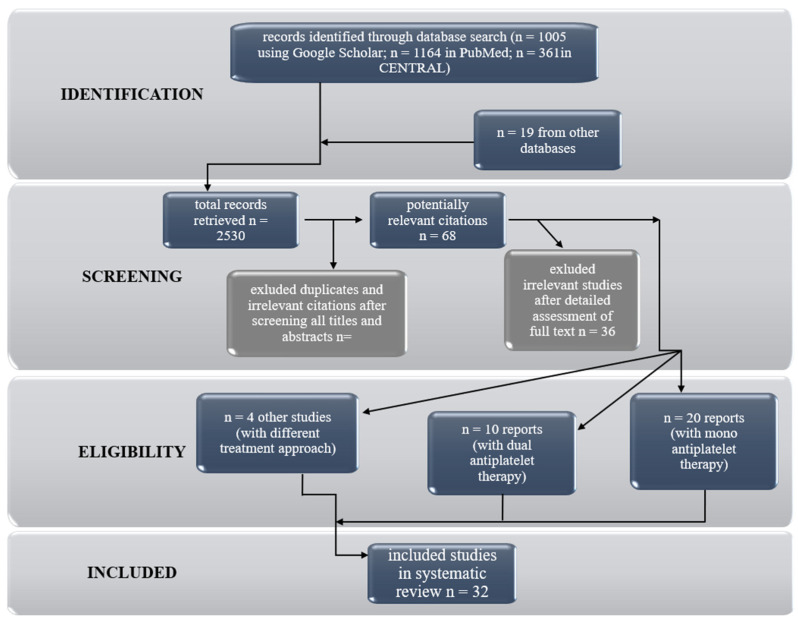
The flow chart of the studies selection.

An investigation of recently published literature was conducted based on PRISMA [10]. Briefly, a database search (date of search 2 November 2021) including PubMed, CENTRAL, and Google Scholar databases. The following keywords were applied: “ischemic stroke”, “acute ischemic stroke” in combination with “antiplatelet drugs” “platelets function monitoring”. References of retrieved studies were searched manually for additional studies and reviews. Reviews were also considered by sources of citations of relevant studies and interpretation of their results. Duplicate and multiple citations and reviews not containing any relevant information were excluded. Eventually, 32 original reports directly related to the importance of monitoring platelet function during antiplatelet treatment after IS were considered eligible for inclusion in the review. The articles were divided into categories according to the treatment approach: dual or monotherapy. A detailed flow chart of the studies selection is shown in Figure 1.

## 3. Results and Discussion

### 3.1. Monitoring of Platelet Function during Dual Antiplatelet Therapy—Aspirin and Clopidogrel Together

Recent AIS guidelines recommend the administration of both antiplatelet drugs over a 21-day period and then sticking to one of these drugs [1]. We compared the studies with different treatments, such as using only ASA or clopidogrel, or a combination of these antiplatelet drugs in patients with AIS.

Li et al. [11] determined the interactions between platelet function testing the formation of plaque (primarily unstable) in the carotid artery, which is detected using ultrasound in patients with AIS. In this study, patients were treated with antiplatelet drugs (ASA—dose range: 100–300 mg or clopidogrel—dose range: 150–300 mg loading dose, maintenance 75 mg, or a combination of both) and hypolipidemic drugs (atorvastatin or rosuvastatin). Clopidogrel has been used in patients who have had problems with the administration of ASA, while dual antiplatelet therapy (DAPT) was given to patients who had a progressive stroke. After admission, the patient underwent a TEG (about 3 days from the start of antiplatelet therapy) and other routine tests, such as lipid profile, fibrinogen, international normalized ratio, prothrombin time, and partial thromboplastin time after activation. Research has shown that it is possible to determine the abnormal clotting process and higher platelet reactivity, which in turn was associated with the production of jugular plaques, especially unstable ones. As well as the unstable plaque type—the heterogeneous and echoing plaque was associated with over-activity of fibrinogen. Based on the obtained results, the researchers concluded that in patients with IS, plaque formation in the carotid artery is associated with abnormal clotting and that platelet activity additionally leads to unstable plaque formation. The authors did not observe any significant differences in the results of TEG in relation to the applied antiplatelet therapy [11].

Rosafio et al. [12] designed an observational study that assess the response to antiplatelet therapy. Researchers analyzed platelet reactivity using Multiplate in AIS patients treated with antiplatelet therapy (ASA 100 mg daily or clopidogrel 75 mg daily or a combination of both). An attempt was made to determine the differences between patients who had received antiplatelet therapy before the onset of stroke and those who did not use antiplatelet drugs in the context of response to therapy. Platelet function was determined 24–48 h after admission to the hospital in the first group, and 7–10 days from the start of treatment in the second group. This study also analyzed CYP2C19 polymorphisms by genotyping TaqMan SNPs compared to the etiological subtypes of stroke (e.g., small artery disease, large artery atherosclerosis, cardiogenic embolism) and response to clopidogrel. It was observed that the response to ASA treatment was more frequent in patients starting treatment during hospitalization than in patients receiving this antiplatelet therapy earlier. In the overall IS population, it was found that patients responded better to ASA treatment than to clopidogrel or DAPT. Moreover, in both studied groups, resistance to clopidogrel treatment was significantly more frequent than to ASA. The investigators failed to identify significant differences in the response to all variants of antiplatelet therapy between the etiological subgroups. The authors conclude that platelet function testing could be potentially useful in monitoring the biological effect of antiplatelet agents in this group of patients [12].

Shao et al. [13] compared three platelet function tests such as LTA (AA and ADP), TEG (AA and ADP), and PFA (EPI and P2Y) among themselves in the context of IS patients receiving antiplatelet therapy. After treatment with ASA 100 mg daily or clopidogrel 75 mg once daily or a combination of both drugs for at least 5 days. In addition to the above-mentioned tests genotyping was also performed (the CYP2C19 genotype was analyzed). After the test was performed, it was observed that all platelet function tests were effective in determining the effect of ASA (however, there was only agreement between the LTA and TEG methods). LTA and PFA methods were better in assessing the effect of clopidogrel (but the correlation between the tests was weak). LTA and PFA were consistent with genotyping. Therefore, by using LTA and TEG (AA), the results can be combined by monitoring the effect of ASA. The PFA-EPI test can be used to determine resistance to treatment. For clopidogrel therapy, it is better to use a different method than TEG-ADP. In order to adjust antiplatelet therapy, it is worth combining CYP2C19 genotyping with the LTA-ADP or PFA-P2Y test [13].

Bath et al. [14] conducted a study as part of a larger TARDIS study that assessed the safety and efficacy of antiplatelet therapy in patients with transient ischemic attack (TIA) or AIS. TIA is a common type of stroke. The blockage of blood flow through the brain occurs over a period of time. Therefore, TIA does not cause permanent brain damage, but it should be borne in mind that in some time it may lead to an IS [15]. In contrast, this study looked at the effect of antiplatelet therapy (when patients were taking them at the start of the study) on platelet function as assessed by measuring the expression of P-selectin found on the platelet surface. Prior to randomization, patients received antiplatelet therapy such as ASA or clopidogrel, or a combination of these drugs, or ASA with dipyridamole. The study also included people who had not taken antiplatelet drugs prior to randomization. The expression of P-selectin on the platelet surface was measured by flow cytometry before and 7 days after randomization, which is important, the method does not determine the effect of dipyridamole. Researchers observed that ASA reduced the expression of AA-stimulated P-selectin in the group of patients with AIS or TIA, while the P2Y12 inhibitor had decreased expression of ADP-stimulated P-selectin. On the other hand, DAPT with ASA and clopidogrel increased the levels of AA- and ADP-stimulated P-selectin compared to monotherapy with these drugs. In conclusion, it was found that the measurements of P-selectin depend on the antiplatelet therapy used, and a small percentage of patients turned out to be refractory to treatment with clopidogrel [14].

Yi et al. [16] designed a study to evaluate the effect of treatment in two groups of patients: clopidogrel + ASA and ASA alone on neurologic deterioration, platelet activation and other short-term outcomes. Five hundred and seventy-four patients were enrolled in this study. In all patients, the stroke was caused by large-artery atherosclerosis. ASA patients received 200 mg/day daily for 30 days, then 100 mg/day thereafter; whereas the clopidogrel + ASA group was treated with 200 mg ASA + 75 mg clopidogrel continuing for 30 days and 75 mg/day clopidogrel thereafter. Platelet aggregation was assessed before receiving ASA or clopidogrel and on day 30 of therapy. Aggregation was measured by optical platelet aggregometry (arachidonic acid (5 mM) and ADP (10 µM)). The obtained results suggested that combined antiplatelet therapy (clopidogrel + ASA) reduced the incidence of recurrent IS compared to monotherapy (ASA). Moreover, platelet aggregation was lower in patients receiving the combination of clopidogrel and ASA than in ASA alone [16].

Yi et al. [17] assessed the association between clopidogrel resistance and early neurological deterioration in their prospective, observational, two-center study, and analyzed the stratification of the efficacy of clopidogrel alone and clopidogrel with ASA in preventing early neurological deterioration. The study population was divided into two groups: (1) patients received clopidogrel alone (*n* = 144) and (2) patients received clopidogrel and ASA (*n* = 231). There were two time points at which was measured platelet aggregation- before the initial dose of clopidogrel and after the 7–10 days. Platelet aggregation was measured by light transmittance aggregometry. Two agonists were used to evaluate platelet activity: 0.5 mM arachidonic acid and 10 μM ADP. There were 153 (40.8%) patients clopidogrel resistance and 222 (59.2%) patients clopidogrel sensitive. The obtained results indicate the frequency of early neurological deterioration was significantly lower in patients receiving clopidogrel plus ASA than patients receiving clopidogrel alone [17].

Lu et al. [18] investigated the relationship between vitamin D (as measured by an electrochemiluminescent immunoanalyzer) and platelet activation which was determined by quantifying CD62p on platelet (as measured by a flow cytometer) and soluble P-selectin in plasma ((as measured by an enzyme immunoassay) and the occurrence of resistance to antiplatelet therapy in patients with IS. One to two weeks after starting antiplatelet therapy with ASA and clopidogrel (in accordance with Chinese guidelines—loading doses were 300 mg of clopidogrel and ASA and subsequent doses of 75 and 100 mg daily, respectively), the above parameters and platelet aggregation using turbidimetric aggregometry were assessed (Techlink biomedical device, Bozeman, MT, USA). Based on the results, it was observed that resistance to P2Y12 inhibitor was more common in patients with IS than ASA. For P-selectin, no significant difference was observed in the absence or presence of resistance to the thienopyridine derivative. The authors summarizing their research concluded that low vitamin D levels but also increased CD62p expression were independent risk factors for clopidogrel resistance in patients with IS [18].

In the above studies, the main difference is the use of other methods of assessing platelet function, because Li et al. and Shao et al. used TEG for the assessment of platelet function, but in other studies LTA, PFA, and genotyping were used. A method of testing platelet function, i.e., the optical platelet aggregometry (PAP-4 aggregometer), appeared in the research of the Yi team. In contrast, Rosafio et al. used Multiplate in their research, and in the work of Bath et al. and Lu et al., a flow cytometer was used. Each of these methods has its advantages and disadvantages, but it was possible to monitor the function of platelets, which allowed to determine resistance to treatment [12,14,17,18] or the reason to produce unstable plaques. When comparing the dosing of antiplatelet drugs, some similarities can be noticed, details in Table 2, namely in all studies the maintenance dose of clopidogrel was 75 mg, and ASA doses were in the range of 100–300 mg (an exception is the work of Bath et al., where subsequent doses were reduced to 75 mg). Due to the use of ASA and dipyridamole (2 × 200 g/day) in patients after AIS or TIA. The time points in the presented study were the measurement before and after the initiation of therapy, respectively, in the work of Rosafio et al. 24–48 h after admission and 7–10 days after the first administration of the drug. In the studies of the Yi team [16,17], the time points were similar—they were measured before and 7–10 days after the start of treatment. In the study by Bath et al. however, measurements were taken before and 7 days after randomization, and in the work of Li et al. platelet reactivity was measured 3 days after the initiation of antiplatelet therapy. In the analyses of Lu et al. and Shao et al., 1–2 weeks and 5 days from the start of administration of drugs reduced platelet aggregation, respectively [11,12,13,14,15,16,17].

### 3.2. Monitoring of Platelet Function during Dual Antiplatelet Therapy—First Aspirin Then Clopidogrel

This different therapeutic approach is presented in the three studies presented below, patients were taking DAPT, but ASA was used first, and then IS patients were administered clopidogrel as secondary prevention of stroke. Ciolli et al. [19] determined the reactivity of platelets after the procedure performed during endovascular treatment—carotid artery stenosis in IS patients. This study was used to determine the predictability of functional outcomes in the study group, including stent thrombosis and hemorrhagic transformation over a period of 90 days. Platelet activation was monitored using the Multiplate (ASPI test, ADP test, TRAP test) within 10 days of the onset of stroke. In this analysis, ASA and clopidogrel were used as antiplatelet drugs before and after the procedure. Patients were identified as responders or non-responders to treatment with ASA and clopidogrel. From the obtained values of the ASPI/TRAP ratio, it was found that resistance to ASA was independently the basis for predicting poor outcomes and mortality. Based on this, a tendency to lower hemorrhagic transformation and multiple stent thrombosis was also observed. However, in the case of clopidogrel resistance, the multivariate analysis showed more favorable results. The authors concluded that the analysis of platelet function may be useful in the care of stroke patients [19].

In turn, Sternberg et al. [20] in their study analyzed the antiplatelet effect of clopidogrel therapy in IS patients who had previously been treated with ASA. TEG, VerifyNow, and LTA were used for the analysis of platelet reactivity at the time points: prior to clopidogrel administration and 26 and 64 h after drug administration. Before the clopidogrel therapy, it was observed that all platelet reactivity tests showed a poor response to ASA. On the other hand, 26 h, and 64 h after the administration of clopidogrel, the devices registered a marked inhibition of platelet function. There were also more patients who responded poorly to treatment at 26 h (also depending on the type of test) than at the 64 h time point. Moreover, in the case of high baseline platelet reactivity in IS patients and the increased effect of P2Y12 inhibitor, there was a potential for improvement in treatment outcomes. The authors concluded that the poor response of clopidogrel treatment depends on the antiplatelet monitoring device used [20].

Wiśniewski et al. [21] conducted a prospective, single-center, observational study. The investigators focused on the assessment of HTPR during clopidogrel treatment depending on the size of the acute and chronic ischemic changes in the brain. Seventy-four patients with IS participated in this study. All patients received 150 mg of ASA after excluding hemorrhagic stroke. The next day, they started treatment with 75 mg of clopidogrel as secondary prevention of stroke. Platelet reactivity was measured with the Multiplate test at two time points: 6–12 h and 48 h after a dose of clopidogrel. It has been shown that a decrease in platelet reactivity was observed in the subgroup with mild severity of chronic vascular lesions. However, in subgroups with moderate and severe chronic vascular lesions, it turned out there was no significant decrease in platelet reactivity. In addition, the researchers were used multivariate regression models to report in this study unfavorable dynamics of platelet reactivity alone and combined with a high initial value of platelet reactivity as independent predictors of higher risk of a significant ischemic infarct volume [21].

All presented articles were characterized by a different device for the analysis of platelet function, in the case of Ciolli et al. and Wiśniewski et al., it was multiplate, while Sternberg’s used TEG, VerifyNow, and Chrono-Log. The method used to assess the activity is of great importance in terms of the obtained results, what is known from the study by Koziński et al. [22]. The first drug used in these studies was ASA, the dosage of this drug differed significantly (Ciolli et al.—dose range: 250–100 mg; Sternberg et al.—dose range: 81–325 mg; Wiśniewski et al.—150 mg). The second antiplatelet drug was clopidogrel—75 and 300 mg appeared in both articles (Ciolli et al. and Sternberg et al.) but in the study by Wiśniewski et al.—75 mg. The time in which platelet function was measured in the work of Ciolli et al., Sternberg et al., and Wiśniewski et al. was very different—see Table 3 for details.

### 3.3. Monitoring of Platelet Function during Monotherapy—Aspirin or Clopidogrel

Despite the benefits of DAPT in IS patients, many clinicians use ASA or clopidogrel therapy, separately. Marquardt et al. [23] assessed the platelet function in patients after IS and the relationship between platelet activation and inflammation (inflammatory markers such as C-reactive protein, fibrinogen, and leukocytes). In this study, patients were treated with ASA, clopidogrel, or anticoagulants, and the method of measuring platelet activation was flow cytometry (CD62p and CD63 expression). The above-mentioned parameters were measured 24 h after IS and after 2, 3, 5, 7, 10, 14, 28, 45, and 90 days. Flow cytometry has been observed to be a reproducible method of assessing platelet activation markers with low intra-assay variability. In addition, it has been determined that CD62p expression is downregulated in the first week after stroke, and CD63 expression is upregulated for at least 3 months after IS. In the case of inflammatory parameters, no relationship was found with the analyzed parameters of platelet activation. Researchers concluded that the number of neoantigens increased after stroke and CD62p expression decreased, however, CD63 expression remained high during secondary prevention [23].

A similar study was conducted by Yip et al. [24]. Using the same device, they investigated changes in platelet activation in acute phase IS patients. Only CD62p expression was analyzed at the 48 h and 7-, 21-, and 90-days post-stroke time points. Patients were treated with ASA, clopidogrel (if they were ASA intolerant), or warfarin (if there was a clot in the carotid artery, but heparin was given first). The main results of this analysis were that CD62p expression was significantly higher in AIS patients (compared to the risk control group and healthy volunteers). CD62p expression decreased significantly, but this expression was still significantly higher in patients after IS. Antiplatelet therapy with clopidogrel reduces CD62p expression after a period of 3 months. Based on the above results, researchers concluded that platelet activation occurs in the acute phase of a stroke and declines over time after IS. Moreover, CD62p expression remained significantly higher in convalescent and chronic stroke patients compared to healthy volunteers. In addition to comparing antiplatelet drugs, clopidogrel compared to ASA causes a significantly decreased expression of CD62p during therapy and chronic period of IS [24]. In both articles, the authors did not mention the dosages of antiplatelet or anticoagulant drugs. Interestingly, in both studies, significant differences in CD62p expression were observed depending on the antiplatelet drug used—it was smaller in patients treated with clopidogrel.

Tobin et al. [25] undertook the analysis of platelet function during ASA or clopidogrel treatment in patients after IS or TIA. Platelet reactivity was assessed before and after (14 and 90 days) change of treatment. The PFA-100 C-EPI test was found to detect HTPR in patients after IS or TIA taking ASA, while with the PFA-100 C-ADP test it is not possible to reliably capture the effect of the P2Y12 inhibitor. Lower levels of neutrophil–platelet complex formation were observed after switching from ASA monotherapy to clopidogrel (within 14 days later), but not after 90 days. Interestingly, the change in antiplatelet therapy also reduced parameters including complete blood count after 14 days and a decrease in mean platelet volume at both time points. By changing the cross-sectional definition of HTPR to the longitudinal definition, the authors concluded that PFA-100 measurements could monitor the ex vivo response to ASA [25].

Depta et al. [26] assessed platelet reactivity tests used in secondary prevention in patients with TIA or IS. In this study, platelets were monitored by optical aggregometry (PAPS-4 aggregometer). This method was used to determine resistance to antiplatelet drugs, such as ASA or clopidogrel, and then the treatment was modified by increasing the dose or adding another drug (sometimes even switching from ASA to clopidogrel). It has been observed that changes in antiplatelet therapy following platelet reactivity testing may be integrated with an increased incidence of death, bleeding, or ischemic events compared to the group of patients who did not receive modified antiplatelet therapy. Based on the obtained results, the authors concluded that the platelet reactivity test is not helpful in optimizing the clinical results [26].

Antiplatelet therapies were modified in the two studies described above. In the study by Marquardt et al. and Yip et al., only one method (flow cytometry) was used to determine platelet reactivity. In the analysis by Tobin et al., two methods were used to assess platelet function, while Depta et al.’s work was based only on the PAPS-4 aggregometer. Many time points were used in the studies by Marquardt et al. and Yip et al.—10 and 4, respectively—details in Table 4. In Marquardt et al., scientists also measured CD63, so they could determine that CD63 expression increases after stroke and remains high compared to CD62p, whose expression declines over time. Interestingly, in the article by Depta et al., the dosage of the P2Y12 inhibitor was even above the commonly used dose of 75 mg.

### 3.4. Monitoring of Platelet Function during Monotherapy—Aspirin

There are studies in the literature in which patients were treated only with ASA. Wiśniewski, Filipska et al. [27] analyzed the platelet function measured in patients with IS treated with ASA (depending on etiopathogenesis) in the context of patients’ status (clinical and functional) as well as prognosis (early and late). Patients were divided into a group with large or small vessel disease. Platelet function was assessed 24 h after the onset of stroke using LTA and Multiplate. These methods were used to determine the resistance to ASA. Researchers observed that patients with large vessel disease and severe neurological deficits had high platelet function. In addition, the occurrence of resistance to ASA is associated with a more frequent possibility of a severe clinical condition. It was found that the activity of platelets dependent on the etiopathogenesis of stroke affects the functional state in the early phase as well as independently of the etiology. High platelet reactivity (during antiplatelet therapy IS) is associated with an unfavorable late prognosis [27].

In another research paper Wiśniewski, Sikora et al. [28] analyzed the effect of high platelet reactivity during ASA treatment (as above, i.e., at a dose of 150 mg) on ischemic changes (acute ischemic focus volume and chronic vascular changes) in patients after stroke caused by large or small vessel disease. In addition, the patients underwent Doppler examination of the carotid arteries and MRI of the head with volumetric evaluation 5 and 2–5 days, respectively, after the onset of cerebral ischemia symptoms. It has been observed that if there is high platelet activity in patients with bigger vascular occlusion, the bigger is the ischemic focus in the brain. It has also been noted that large arterial atherosclerosis may be an important factor in platelet activation. Based on the analysis of the study results, the researchers concluded that patients after a stroke caused by large vessel disease have high platelet reactivity during antiplatelet therapy. This was associated with a larger area of acute ischemic lesions and the extent of chronic ischemic lesions in the brain. The etiopathogenesis of IS is important in the context of the existence of significant relationships between platelet reactivity and the volume of acute and chronic changes in the brain [28].

On the other hand, Karepov et al. [29] analyzed patients treated with ASA a month after the first IS in terms of the relationship between blood lipid components and platelet function. Platelet function was analyzed using PFA-100. The authors concluded that ASA may not only affect the cyclooxygenase pathway [29]. Yi et al. [30] conducted a study in a population of Chinese post-IS patients treated with ASA to determine non-response to treatment and the frequency of events such as recurrent stroke, myocardial infarction, peripheral arterial disease, and even death. A BioData PAPS-4 platelet aggregometer was used for the analysis of platelet function, which was measured 7–10 days after the start of ASA administration. The observation period for the above-mentioned events was 1–2 years. In this study, it was observed that the majority of patients responded well to ASA while less responding to treatment accounted for a smaller percentage (in this group, there were fewer patients with semi-resistance than patients resistant to acetylsalicylic acid). Based on the obtained results, it was found that the presence of diabetes and high LDL values in patients may increase the probability of developing resistance to ASA. Additionally, poor responders may have a greater risk of another stroke, heart attack, peripheral arterial disease, or death from any cause [30].

Another prospective, multicenter cohort study Yi et al. [31] assessed if prior statin and aspirin treatment could reduce the neurological deterioration of patients with IS. Moreover, the effect of prior statin and ASA treatment on platelet activity was investigated. All patients received ASA, details about the dosage of drugs in Table 4. Patients were received atorvastatin or rosuvastatin if LDL-C was above 70 mg/dL or if the patients’ origin of IS or TIA was presumed to be atherosclerotic. The patients were divided into four groups: (1) prestroke alone statin use group—126 (11.2%), (2) prestroke alone statin use group—137 (12.2%), (3) prestroke statin + ASA use group—231 (20.6%), (4) without prestroke statin or ASA use group—630 (56%). Platelet aggregation was measured by LTA with two agonists—arachidonic acid (5 mM) and ADP (10 μM)—in two time points (on admission and during days 7–10 after admission). The results indicate that platelet aggregation in the first time point was significantly lower in patients with pre-existing statin or ASA. Furthermore, the group of patients with prestroke statin and ASA treatment had a significantly lower incidence of neurological deterioration compared with those without treatment. These findings clearly indicate that concomitant statin and ASA use before a stroke occurs is associated with lower neurological deterioration and platelet activity [31].

Tsai et al. [32] in their prospective cohort study focused on the assessment of the difference in platelet activity among patients taking statins before and after AIS. The additional analyzed aspect was if potential statin treatment reduced neurologic deterioration and improved the functional outcome of patients with IS. The patients were divided into two groups: (1) taking statins prior to stroke onset and (2) without pre-existing statins. The second group was further divided—patients who received statins after stroke onset and the non-statin treatment group. All subjects were treated with ASA (100 mg/day). Platelet activity was assessed using platelet activation markers: CD62P and CD63 by flow cytometry at three time points within 48 h after acute stroke and on days 7 and 30. Significantly, lower expression of CD62P and CD63 on platelets was observed in the patients with pre-existing statin treatment compared to the patients without statin on day post-stroke. Moreover, lower expression of CD62P persisted 90 days after the acute stroke. The severity of the initial stroke was relatively low in patients with statin treatment before stroke onset and lower incidences of early neurologic deterioration [32].

Harrison et al. [33] assessed platelet function using aggregometer Biodata-PAP-4, PFA-100, and VerifyNow in terms of reproducibility in patients after IS or TIA treated with ASA, details about dosage in Table 4. ASA resistance was found to be more often detected with VerifyNow-ASA or PFA-100 C-EPI than with LTA. After the same tests were performed in follow-up one year later, the results were similar, but the rates of non-response to ASA were higher with PFA-100 and VerifyNow than with the Biodata PAP-4 aggregometer. Despite the lack of consistency, it has been concluded that a first-time test may be used initially to predict vascular risk, while a one-year test may be useful for longer-term risk prediction. It was also added that the lack of consistency over time in the determination of apparent lack of response to treatment is likely to undermine the ability of these methods to determine the risk of recurrent vascular events [33].

A study designed by Sabra et al. [7] analyzed platelet reactivity measured with Multiplate (ADPtest, ASPItest, and COLtest) in patients treated with ASA after the first IS. This study also analyzed the frequency of high platelet reactivity during treatment after admission to hospital and within 3–5 days of starting ASA therapy (dosage of 300 mg). This evaluation observed that platelet reactivity with each test was higher in patients who had not been treated with antiplatelet drugs before. In healthy subjects, changes were found between patients who were constantly taking ASA and those who had not used this drug before. It was also noticed that a small percentage of patients did not respond adequately to the treatment 3–5 days after the administration of ASA in the hospital. Based on the results, the authors found that many people in the low-dose ASA group showed high platelet reactivity during treatment or drug resistance. The results question the benefit of administering ASA to patients with a laboratory-measured response to treatment when it did not prevent recurrence of stroke [7].

The aim of the study by Kim et al. [34] was to analyze whether HTPR and its changes after consecutive ASA treatments during the acute period of IS were associated with the occurrence of subsequent vascular events. The measurement of the ASA reaction unit was performed using the VerifyNow system in two time points: (1) 3 h after ASA administration, (2) after 5 days of treatment. In the first time point HTPR was 14% and in the second time point 12.3%. This study also looked at early neurological deterioration (END) in three categories: progressive, with new lesions and other causes, on admission and after 5 days. The researchers found that more than half of the patients who initially showed high platelet reactivity to ASA, however, responded normally to antiplatelet therapy after 5 days. As has also been recognized, determining platelet function may enable the prediction of END. Summarizing their results, the authors concluded that high thrombocyte function over time is independently associated with END in IS patients, and it is more beneficial to perform a series of platelet function measurements. The authors of this study suggested that HTPR during the acute stage of IS increases the risk of subsequent vascular events. Moreover, measurements of ASA response units following a stroke episode may identify patients with AIS who are at a higher risk of vascular events [34].

Jastrzębska et al. [35] analyzed the usefulness of laboratory methods such as the platelet function test (Multiplate), enzyme immunoassay (TXB2), and immunoturbidimetric (vWF) methods for assessing the response to ASA in everyday practice. The study group consisted of patients with AIS treated with ASA at a dose of 150 mg or 300 mg daily. It is also worth mentioning that in the case of increasing the dose of ASA after the next 7 days, tests were repeated. If resistance to ASA was detected, the drug was switched to clopidogrel. Additionally, the neurological deficit index (National Institutes of Health Stroke Scale—NIHSS, Bethesda, MA, USA) was also determined. Researchers noticed that most patients did not respond to the higher doses of the drug, and the response to antiplatelet treatment with ASA was not affected by the plasma concentration of TXB2. In the case of the cofactor vWF, an association was observed between the results from Multiplate and the concentration of CRP (in the group of patients resistant to treatment). CRP was also correlated with platelet reactivity and the level of fibrinogen. Significantly lower levels of HDL cholesterol and higher NIHSS were observed in the group of patients refractory to treatment with ASA. The authors concluded that patients after IS are characterized by an increased concentration of CRP and fibrinogen, increased leukocytosis, and an increased percentage of neurological deficit, with a negative role of diabetes. Researchers suggest using the Multiplate because it reflects the clinical condition of a patient with a stroke and thus it is possible to modify the therapy by switching to clopidogrel [35].

Lee et al. [36] examined whether the addition of cilostazol to acetylsalicylic acid, an antiplatelet therapy in patients with IS, reduces the incidence of ASA resistance in this population. Prior to randomization, patients enrolled in the study were taking (at least 14 days) acetylsalicylic acid 100 mg per day. The patients were divided into two groups: (1) cilostazol in a dose of 100 mg twice a day was added ASA, (2) placebo was added to the ASA therapy for one month. Platelet reactivity was measured with the VerifyNow ASA test before and after 4 weeks of therapy. It was observed that administration of cilostazol did not decrease the occurrence of ASA resistance compared to placebo, but the proportion of patients with resistance decreased over time after administration of cilostazol. Although the researchers did not confirm that the combination of ASA with cilostazol reduces the incidence of ASA resistance in IS patients, they found that there is a tendency to use such combination therapy, however, further research is needed [36].

In all 11 studies, the dosage of the antiplatelet drug was diverse. Wiśniewski et al. used a dose of 150 mg of ASA in both analyses, which was similar to the studies by Yi et al., where the dose of ASA was 100–200 mg [30,31]. In addition, there were two more studies in which the dose of ASA was the same—100 mg [32,36], while in the remaining studies the drug was administered in the range of 75–325 mg [29], 75–150 mg [33], 100–300 mg [34], and 150–300 mg [35], the exception is one study where the dose was 300 mg [7]. The time points at which platelet function was tested were also not the same in the articles, details in Table 5. In the context of the methods used to determine the reactivity of platelets in the studies described above, the following appears: LTA [27,28,31], Multiplate [7,27,28,35], Biodata PAP-4 aggregometer [30,33], PFA-100 [29,33], VerifyNow [33,34,36], and flow cytometry [32]. On this basis, most scientists use only one method of assessing the function of platelets (only one study—by Harrison et al.—used as many as three different devices).

### 3.5. Monitoring of Platelet Function during Monotherapy—Clopidogrel

In addition to the analysis of ASA monotherapy alone, there were also several articles in which the study group consisted of patients after IS or TIA who were administered clopidogrel. Varvat et al. [37] investigated the response to a P2Y12 inhibitor after treatment of patients with IS (non-cardioembolic origin) or TIA. The study used platelet reactivity tests such as VASP, CD62p (flow cytometry), and LTA. These measurements were taken 5–8 days after starting clopidogrel therapy. Transmission electron microscopy (TEM) was used on patients with discrepant results of the above-mentioned methods and an inadequate response to the antiplatelet drug, which was administered at a dose of 75 mg for 24 h (clopidogrel was assigned to patients if found to be preferable to acetylsalicylic acid). The authors found that the low correlation between the tests may be due to the mechanism of device operation in terms of platelet activation. LTA works based on changes in the structure of GPIIb/IIIa glycoproteins, while VASP is based on the analysis of the P2Y12 receptor, and CD62p assesses CD62p externalization. The TEM images show the ultrastructure of the platelets, which reflects the activation phase of the platelets in response to ADP. Hence, the use of TEM in specific clinical cases may be important when there is no overlap between other methods of assessing platelets, including the effectiveness of antiplatelet therapy [37].

A similar study was the analysis by Bagoly et al. [38], however, they conducted a study comparing the innovative ADP platelet function test (PGE) with the conventional ADP test—Chrono-Log, VASP—flow cytometry and VerifyNow P2Y12 in patients with IS who were administered clopidogrel, details about treatment in Table 6 It was noted that the ADP platelet reactivity method specifically inhibits the P2Y12 receptor, therefore, ASA has no influence—the response to clopidogrel can be monitored in patients treated with DAPT. On the other hand, the best correlation was between the new platelet aggregation test and the VASP test. Researchers developed a new platelet reactivity assay that is specific for the P2Y12 receptor, which is suitable for clopidogrel treatment. Compared to the conventional method, this method is not sensitive to ASA used in patients [38].

Qiu et al. [39] investigated the use of the platelet function test—flow cytometry as a method to predict clinical outcomes in patients with AIS who were taking clopidogrel at a daily dose of 75 mg. Flow cytometry is based on the phenomenon of hydrodynamic focusing—blood that has been diluted in a slow flow surrounded by a shielding solution flows faster to the recording systems. Single cells are irradiated by a laser. The detectors record changes in the electrical impulse caused by each of the cells. To analyze platelet activation, ADP-PAg-induced platelet aggregation and markers such as CD62p, CD63 and PAC-1 were determined (measurements were made before and 7 days from the start of clopidogrel therapy). It has been observed that the presence of high platelet reactivity in response to treatment predicts unsatisfactory clinical outcomes after IS within one year. On this basis, the HTPR phenomenon detected by cytometry can be used to reveal IS patients with an increased probability (12 months) of poor results of treatment with the P2Y12 inhibitor—clopidogrel [39].

Meves et al. [40]—in their prospective observational study—evaluated in AIS patients the prevalence and risk factors for clopidogrel HTPR. During hospitalization, all patients received 75 mg clopidogrel daily. Platelet aggregation was analyzed 48 h after therapy was either initiated or continued, using Multiplate. Forty-four percent of patients were clopidogrel HTPR. The researchers showed diabetes mellitus and higher HbA1c values as risk factors for clopidogrel HTPR, wherein multivariate regression analysis revealed that diabetes mellitus more than doubled the risk [40]. On the other hand, Rath et al. [41] established the prevalence of HTPR in the hyper-acute stroke phase at 8–24 h after the intake of clopidogrel 300 mg. In this prospective cross-sectional study, 219 patients with ischemic stroke and TIA were included. Platelet functions were analyzed using the VerifyNow system, 8–24 h after clopidogrel treatment. The obtained results indicate that 28.8% patients exhibited HTPR after the intake of clopidogrel. Rath et al. suggested that recognition of HTPR acute phase after stroke may be an important step toward interventions that may further minimize the early recurrent stroke risk [41].

Two studies [37,38] in this section used the VASP test to analyze platelet reactivity—see Table 6 for detailsApart from this method, Varvant et al. also tested the expression level of CD62p on the flow cytometer, other methods used were LTA and the novelty of TEM to resolve inconsistencies between tests. Qiu et al. also used a flow cytometer in their research and measured the level of CD63 and PAC-1 expression. Bagoly et al. used VerifyNow and Chrono-Log. VerifyNow was also used in the study by Rath et al., while in the analysis by Meves et al. platelet function was determined using the Multiplate. The clopidogrel dose in most articles was 75 mg [37,38,40], only Rath et al. used 300 mg (these patients were also given 300 mg ASA). The authors presented in this section stated that different mechanisms of operation of individual tests are the basis for a low correlation between tests and in situations requiring resolution it is worth using TEM and a new method—the ADP test (PGE1) is a very good option for clopidogrel therapy.

### 3.6. Monitoring of Platelet Function—Other Studies

Coignion et al. [42] assessed the importance of platelet aggregation testing in the identification of stroke etiology. The efficacy of antiplatelet drugs was assessed using a multi-plate analyzer with two agonists: arachidonic acid and ADP. Resistance to antiplatelet drugs was found in 18.5% of participants. There were no significant differences in the mechanism of stroke depending on antiplatelet drug efficacy. The authors of this study concluded that patients sensitive to antiplatelet drugs had less severe initial stroke severity according to mean NIHSS. Moreover, they suggested main indicators for antiplatelet drug resistance were a worse control of diabetes and higher baseline levels of inflammatory factors [42].

Cha et al. [43] assessed the effect of high residual platelet activation after ADP stimulation on the frequency of recurrent cardiovascular events and mortality in patients with AIS treated with antiplatelet drugs after one year. In this observational, referral center cohort study, all subjects received antiplatelet therapy: ASA alone—300 mg on the day of admission followed by 100 mg of ASA on subsequent days or dual antiplatelet agents—300 mg of clopidogrel + 100 mg of ASA on the day of admission and maintained 75 mg and 100 mg daily (see Table 7 for details). Platelet aggregation was assessed by optical platelet aggregometry on day 5 after the start of hospitalization. 70% or greater in ADP-induced platelet aggregation was defined as high residual platelet activation in this trial. Primary endpoint: all causes of death, myocardial infarction, and stroke at the 1-year follow-up. The primary endpoint was noted in 11.3% of patients. More often in the patients with high residual platelet activation (16.7%) than in those without (9.7%). The scientists concluded that the presence of high residual platelet activation after ADP stimuli is associated with worse outcomes of patients after AIS [43].

### 3.7. Recommendations from Clinical Trials

Treatment of atherothrombosis with clopidogrel in high-risk IS or TIA was associated with an increased risk of life-threatening or major bleeding with no reduction in major vascular events at 18 months [44]. On the other hand, the prevention regimen for effectively avoiding second strokes trial compared ASA plus extended-release dipyridamole to clopidogrel and found no difference between the two groups in recurrent stroke rate [45]. However, more intracranial hemorrhage was observed in the aspirin-dipyridamole group. The secondary prevention of small subcortical strokes trial showed that, among patients with recent symptomatic lacunar stroke, the addition of clopidogrel to ASA resulted in an increased risk of bleeding and death without a reduction in risk of recurrent stroke after a mean follow-up of 3.4 years [46]. More recently, the clopidogrel in high-risk patients with acute nondisabling cerebrovascular events trial investigated whether DAPT may be beneficial in the short-term, as opposed to over the long-term, in patients with recent minor IS or TIA [47]. In this study, patients were randomized to treatment with ASA and clopidogrel for 21 days post-stroke or to ASA plus placebo for 21 days post-stroke. Treatment was initiated within 24 h of the qualifying event. Recurrent stroke occurred in 8.2% of patients in the DAPT, as compared with 11.7% of those in the ASA group [48]. In this study, 4881 patients were enrolled, and IS was observed in 4.6% of the DAPT compared with 6.3% of the ASA group. The benefit of DAPT was greater in the first 30 days than at days 31 to 90, and the risk of major hemorrhage was lower in the first 7 days than from days 8 to 90.

Unlike CHANCE and POINT, The Acute Stroke or Transient Ischemic Attack Treated with Ticagrelor and ASA for Prevention of Stroke and Death (THALES) trial examined the benefit of DAPT with short-term ASA and ticagrelor for 30 days after stroke. Eligible patients had a mild-to-moderate acute non-cardioembolic IS or high-risk TIA or symptomatic intracranial or extracranial arterial stenosis. Randomization occurred within 24 h of symptom onset, and all patients received a loading dose of ASA and either a loading dose of ticagrelor 180 mg or a “loading dose” of placebo as soon as possible after randomization. ASA and ticagrelor or placebo were continued for 30 days. A total of 11,016 patients were included in THALES. The primary endpoint of composite stroke or death occurred in 5.5% of patients in the DAPT group and 6.6% in the ASA group. Severe bleeding occurred in 0.5% of patients in the DAPT group and in 0.1% in the ASA group [48,49].

Based on the positive results of CHANCE and POINT, the stroke community has largely adopted the guideline-concordant use of DAPT with ASA and clopidogrel for 21 days post-stroke. In 2019, the American Stroke Association guidelines were updated to include a highest-level recommendation that “in patients presenting with minor non-cardioembolic IS who did not receive IV alteplase, treatment with DAPT (ASA and clopidogrel) started within 24 h after symptom onset and continued for 21 days is effective in reducing recurrent IS for a period of up to 90 days from symptom onset [1]”. As a result of this recommendation, short-term DAPT with ASA and clopidogrel after a qualifying TIA or minor ischemic stroke has become standard of care.

### 3.8. Summary

In both studies, Yi et al. [16,17] found that DAPT is safer and more effective in the context of recurrent stroke as well as worsening of the neurologic condition. Interestingly, in the study by Shao et al., after comparing the methods, the authors concluded that resistance to treatment is best determined by the PFA-EPI method, while in the case of adjusting the therapy with antiplatelet drugs, it is best to combine two methods, i.e., genotyping and LTA or PFA (ADP and P2Y test, respectively). In studies investigating DAPT, Rosafio et al. [12] suggested that monitoring of platelet function could be a useful tool in assessing the effect of antiplatelet drugs in patients with IS. In another study, Shao et al. [13] demonstrated the effectiveness of various methods of monitoring platelet function. However, different methods were appropriate depending on the antiplatelet therapy used. Bath et al. [14] indicated that P-selectin expression is variable depending on the antiplatelet therapy used.

In a group of trials, where the DAPT approach uses firstly ASA then clopidogrel, Ciolli et al. [19] concluded that the analysis of platelet function may be useful in management of patients after IS. Sternberg et al. suggested that the antiplatelet therapy monitoring device was associated with the poor response of clopidogrel treatment. Therefore, the discrepancy in the methods led the authors of the first and third study to conclude that platelet monitoring may be useful (in the work of Wiśniewski et al. it was mainly about the sequential determination of platelet reactivity in the acute phase of IS as a prognostic factor related to the clinical outcome), while the authors of the second study concluded that an inadequate response to a P2Y12 inhibitor in patients with AIS depends on the method used to measure thrombocyte activity.

In patients treated with antiplatelet therapy as monotherapy Marquardt et al. [23] observed that evaluation of the expression of platelet activation markers by flow cytometry was a reproducible method. In their study, Depta et al. [26] concluded that the platelet reactivity test is not helpful in optimizing the clinical results. This is a different conclusion from most studies available. Wiśniewski et al., based on monitoring platelet function, suggested that high platelet reactivity during antiplatelet therapy was related to poor prognosis [27]. Wiśniewski et al. in their research emphasized the importance of the etiopathogenesis of stroke. Harrison et al. [33] compared the monitoring of platelet function with three different methods and concluded that inconsistency between methods is likely to undermine the ability of these methods to determine the risk of recurrent vascular events. In contrast, Sabra et al. [7], who took more measurements, believe that the timing of assessing platelet reactivity for resistance to treatment is important. They question the benefit of administering ASA to patients with a laboratory-measured response to treatment when it did not prevent recurrence of stroke. Kim et al. [34] clearly suggested that it is more beneficial to perform a series of platelet function measurements than a single analysis in the context of assessing platelet function during ASA treatment and process evaluation of early neurological deterioration. Jastrzębska et al. suggested monitoring platelet function by Multiplate because it reflects the clinical condition of a patient with a stroke and thus it is possible to modify the antiplatelet therapy.

On the other hand, Varvat et al. [37] found that the low correlation between methods of measurements is due to the mechanism of device operation in terms of platelet activation. They proposed the use of TEM in specific clinical cases. Bagoly et al. [38] also compared methods of monitoring platelet function and they had developed a new method of platelet reactivity assay, which was not sensitive to acetylsalicylic acid used in patients. Subsequent studies [40,41] concerned the high reactivity of platelets in response to clopidogrel, in the first of them the researchers found that diabetes is one of the main factors contributing to the HTPR phenomenon, in the second, the authors emphasize the importance of quickly determining the occurrence of HTPR because it is then possible to reduce the risk of recurrent stroke [37,38,39,40]. It is worth noting that some of the authors who monitored platelet function did not report the doses of antiplatelet drugs used [11,12,13,23,24,26,40,42].

The results of large clinical trials recommend the use of DAPT in patients with IS while demonstrating a low but noticeable increase in the risk of bleeding. According to the THALES study, severe bleeding occurred in 0.5% of patients in the DAPT group and in 0.1% in the ASA group. On the other hand, there is still a trend in the literature to use the ASA monotherapy approach. The comparison of studies with monotherapy and DAPT shows the superiority of DAPT over the use of ASA and clopidogrel, separately. However, the confirmation of the effectiveness of the applied treatment raises more doubts. As the presented systematic review shows, there are only 32 studies in which the authors monitor platelet function. Unfortunately, without considering the pharmacodynamics of platelets in IS patients treated with ASA (or DAPT of course), it is not certain that they are not in the group that demonstrates poor clinical outcomes. It is known, a proportion of patients treated with ASA demonstrate poor clinical outcomes, which may be attributable to aspirin resistance [50], an increasingly recognized clinical phenomenon. Acetylsalicylic acid resistance is defined by Patrono as an ontogenic limitation of ability to prolongate the bleeding time, inhibition of thromboxane (TXA) biosynthesis, and ability of platelet function inhibition concluding with a deficit of efficacy for preventing cardiovascular incidents [51]. Despite multiple studies, the criteria of resistance have not been clearly defined, and the pathogenesis of this phenomenon is still a subject of debate. Numerous methods are available for laboratory evaluation of platelet function, but these methods evaluate only specific aspects of platelet function [52]. HTPR was defined as a maximum arachidonic acid-induced aggregation of >20% [53]. The frequency of this phenomenon is estimated from 5 to 15%, and even 29%, depending on the application of a testing method [54]. Time passed from hospitalization with simultaneous complaint deficit and cardiovascular complications might lead to disruption of antiplatelet treatment (although all subjects declared regular drug intake). It is considered to be the most frequent trigger of deceptive ASA resistance [55]. Similar conclusions were stated in the study by Schwartz et al. and Tantra et al., where it was proven that medication nonadherence was causing resistance to ASA. The irregularity of drug intake is estimated as 40% among subjects with cardiovascular system diseases [51,56]. A similar prevalence is found in stroke populations [53]. These “aspirin-resistant” patients exhibit an increased risk of vascular events such as myocardial infarction, transient ischemic attack, or stroke. After having a first stroke, the risk of having a major heart incident—such as a heart attack, heart failure, or cardiovascular death—30 days later was 25 times higher in women and 23 times higher in men. One year after a stroke, men and women still had twice the risk of a major cardiac event compared to their peers who had not had a stroke [57]. Pharmacokinetic and pharmacodynamic responses to clopidogrel depend on genetic polymorphisms [58]. The genome-wide association study of clopidogrel response has reported that the CYP2C19*2 genotype (loss-of-function allele), the most common genetic variant, is associated with poor metabolism of clopidogrel with diminished platelet response and poorer cardiovascular outcome. There are several factors associated with clopidogrel resistance: blood glucose level, diabetes, and high systolic and diastolic blood pressure. The incidence of low response or non-response to clopidogrel ranges between 5% and 30% [59,60]. It should be taken into consideration that aspirin-resistant patients may have a high rate of clopidogrel resistance as well.

## 4. Conclusions

Platelets are critical to thrombus formation, contributing as much as 50% of the total thrombus volume. Antiplatelet agents decrease platelet aggregation and, in turn, the size and frequency of thrombolytic emboli (including ASA and clopidogrel together or separately). It is necessary to emphasize that the goal of stroke preventive therapy is to lower the risk—not to make the risk of stroke zero. In patients with TIA or ischemic stroke, antiplatelet drugs can decrease the risk of stroke by 11–15%, if the patients are good responders. However, platelet reactivity is variable, and patients do not respond uniformly to antiplatelet therapy, which influences the outcomes of secondary prevention. Importantly, aspirin-resistant patients may benefit from DAPT with clopidogrel or in addition.

The above literature review shows that the authors do not take a clear position on what antiplatelet therapy should look like in studies that monitor platelet activity. Most of the articles presented indicate the use of platelet monitoring, but in a very short period of time after the initiation of antiplatelet therapy (indicate time points up to 24 h or 5–7 days from the start of antiplatelet therapy). It is worth noting that at least 1/3 of the articles do not declare the exact dosage of antiplatelet drugs. The available literature indicates that there are different antiplatelet treatment strategies in patients with ischemic stroke but results also depend on the applied laboratory method. The method used to assess the activity is of great importance in terms of the obtained results, which is known from the study by Koziński et al. [22]. As indicated in the summary, the results of large clinical trials recommend the use of DAPT in patients with IS, while demonstrating a low but noticeable increase in the risk of bleeding. On the other hand, there is still a trend in the literature to use the ASA monotherapy approach.

Unfortunately, as indicated by the above literature review, apart from the recommendation issued by the American Stroke Association guidelines, 32 studies available in the literature, conducted with platelet activity monitoring, do not provide clear recommendations regarding antiplatelet therapy in the indicated group of patients. In addition to the phenomenon of antiplatelet drug resistance, patients have a different risk of bleeding or thrombosis, therefore, it seems that the best solution might be to monitor platelet activity as secondary prevention. The goal of the personalized approach in secondary stroke prevention is to take the most appropriate medication in the right dose and use the right therapeutic approach for the right patient.

## Figures and Tables

**Table 1:** Advantages and disadvantages of platelet function tests.

Method Name	Advantages	Disadvantages
PFA	No plate preparation stage, conditions similar to natural	Distortion of the results by various factors, e.g., the number of platelets, HCT, vWf, requires pipetting
VerifyNow (optical turbidimetry)	Small blood volume needed for the test, simple operation, fast and standardized procedure	High cost, a specific range of platelets
Multiplate (impedance aggregometry)	Five tests at one time, sample—whole blood, fast procedure, good reproducibility	Semi-automatic device, specified platelet count range, incubation time (hour)
TEG (Thromboelastography)	Sample—whole blood, result—many parameters	Time consuming, test results only with P2Y_12_ inhibitors
ROTEM (rotational Thromboelastometry)	Sample—whole blood, result—multiple parameters, automatic pipetting	Time consuming, other tests needed
LTA (optical turbidimetry)	Recognized method, testing the response to various agonists	No standards, large blood volume needed for the test, long sample preparation time
Flow cytometry	Device for many different measurements, specific, small volume of blood needed for the test	Skilled personnel, high cost, short term reagents, sample preparation, low sensitivity test, short term marker molecules

**Table 2:** Comparison of studies in terms of dosing of antiplatelet drugs during dual antiplatelet therapy.

Study	Object	Population	Antiplatelet Drugs	Platelet Function Assessment Device/s	Time Points
Li, 2019 [11]	Unstable carotid plaque is associated with coagulation function and platelet activity evaluated by thrombelastography	AIS patients (*n* = 407)	ASA (dose range 100–300 mg/day); CLO (dose range 150–300 mg/day loading dose, maintenance 75 mg/day); ASA + CLO (not reported)	TEG	3 days from the start of antiplatelet therapy
Rosafio, 2017 [12]	Platelet function testing in patients with acute ischemic stroke: an observational study	IS patients (*n* = 624)	ASA (100 mg/day); CLO (75 mg/day); ASA + CLO (not reported)	Multiplate; CYP2C19*2 genotyping; CYP2C19*17 Genotyping	24–48 h after admission; after 7–10 days from the start of treatment
Shao, 2020 [13]	A comparison of three platelet function tests in ischemic stroke patients with antiplatelet therapy	IS patients (*n* = 249)	ASA (100 mg/day); CLO (75 mg/day); ASA + CLO (not reported)	LTA (AA and ADP); TEG (AA and ADP); PFA (EPI and P2Y); CYP2C19 genotyping	For at least 5 days
Bath, 2017 [14]	Remote assessment of platelet function in patients with acute stroke or transient ischemic attack	AIS or TIA patients (*n* = 712)	ASA (300 mg/day load and then 75 mg/day); CLO (300 mg/day load and then 75 mg/day); ASA + CLO (not reported); ASA + dipyridamole (typically modified release 200 mg twice daily)	Flow cytometer	Before and 7 days after randomization
Yi, Lin, 2014 [16]	A comparative study of dual versus monoantiplatelet therapy in patients with acute large-artery atherosclerosis stroke	Patients with acute large-artery atherosclerosis stroke (*n* = 574); CLO + ASA patients (*n* = 286); ASA patients (*n* = 288)	ASA (200 mg/day for 30 days and 100 mg/day thereafter); ASA + CLO (200 mg/day + 75 mg/day) from the day of admission and continuing for 30 days and CLO (75 mg/day)	PAP-4D; cytometer	Period of 30 days
Yi, Wang, 2018 [17]	Response to clopidogrel is associated with early neurological deterioration after acute ischemic stroke	IS patients (*n* = 375); CLO patients (*n* = 144); CLO + ASA (*n* = 231)	CLO (75 mg/day); ASA + CLO (200 mg/day + 75 mg/day) for the initial 2 weeks; followed by treatment with CLO (75 mg/day)	LTA; VerifyNow P2Y12	Before and after the 7–10-day treatment
Lu, 2019 [18]	Platelet surface cd62p and serum vitamin d levels are associated with clopidogrel resistance in Chinese patients with ischemic stroke	IS patients (*n* = 230)	ASA + CLO (300 mg/day loading dose and then 100 mg/day + 300 mg/day loading dose and then 75 mg/day)	Flow cytometer; electrochemiluminescent immunoanalyzer	7–14 days from the start of antiplatelet therapy with ASA and CLO

AIS—acute ischemic stroke, ASA—aspirin, ASA + CLO—dual therapy, CLO—clopidogrel, IS—ischemic stroke, LTA—light transmittance aggregometry, PAP-4D—optical platelet aggregometer, PFA—platelet function analyzer, TEG—thrombelastography, TIA—transient ischemic attack.

**Table 3:** Comparison of studies in terms of dosing of antiplatelet drugs during aspirin then clopidogrel treatment.

Study	Object	Population	Antiplatelet Drugs	Platelet Function Assessment Device/s	Time Points
Ciolli, 2021 [19]	Platelet function monitoring performed after carotid stenting during endovascular stroke treatment predicts outcome	IS patients (*n* = 54)	ASA (dose range: 250–1000 mg/day) and CLO (dose range: 75–300 mg/day)	Multiplate	10 days of the onset of stroke
Sternberg, 2013 [20]	Clopidogrel responsiveness in stroke patients on a chronic aspirin regimen	IS patients (*n* = 66)	ASA (81–325 mg/day) and CLO (300 mg/day and the maintenance dose was 75 mg/day)	LTA; TEG; VerifyNow; Chronolog 570VS	Prior to CLO administration, 26 and 64 h
Wiśniewski, Sikora, 2021 [21]	Unfavorable changes in platelet reactivity on clopidogrel therapy assessed by impedance aggregometry affect a larger volume of acute ischemic lesions in stroke	IS patients (*n* = 74)	ASA (150 mg) and CLO from the next day (75 mg/day)	Multiplate (ADP test)	6–12 and 48 h after administration of clopidogrel

ASA—aspirin, ASA, and CLO—first aspirin then clopidogrel, CLO—clopidogrel, IS—ischemic stroke, LTA—light transmittance aggregometry, TEG—thrombelastography.

**Table 4:** Comparison of studies in terms of dosing of antiplatelet drugs during aspirin or clopidogrel treatment.

Study	Object	Population	Antiplatelet Drugs	Platelet Function Assessment Device/s	Time Points
Marquardt, 2002 [23]	Course of platelet activation markers after ischemic stroke	IS patients (*n* = 50); healthy subjects (*n* = 30); risk factor control subjects (*n* = 20)	ASA (not reported); CLO (not reported)	CD62p flow cytometer; CD63 flow cytometer	10 time points—24 h after IS and after 2, 3, 5, 7, 10, 14, 28, 45, and 90 days
Yip, 2004 [24]	Serial changes in platelet activation in patients after ischemic stroke Role of pharmacodynamic modulation	IS patients (*n* = 87); healthy volunteers (*n* = 20); risk factor control subjects (*n* = 33)	ASA (not reported); CLO (not reported)	CD62p flow cytometer	Time points 48 h and 7, 21, and 90 days after the onset of stroke
Tobin, 2013 [25]	High on-treatment platelet reactivity on commonly prescribed antiplatelet agents following transient ischemic attack or ischemic stroke: results from the Trinity Antiplatelet Responsiveness (TRAP) study	IS or TIA patients treated with ASA (*n* = 26); ASA and CLO (*n* = 22)	ASA (300 mg/day at 14 days and 75 mg/day at 90 days); CLO (75 mg/day)	CD62P flow cytometer; CD63 flow cytometer; PFA-100	14 and 90 days
Depta, 2012 [26]	Clinical outcomes using a platelet function-guided approach for secondary prevention in patients with ischemic stroke or transient ischemic attack	IS patients (*n* = 250); TIA patients (*n* = 74)	ASA (not reported); CLO (150 mg/day or higher)	PAPS-4	For 7 days for a short duration after acute coronary syndrome

ASA—aspirin, CLO—clopidogrel, IS—ischemic stroke, PAPS-4—optical platelet aggregometer, PFA—platelet function analyzer, TIA—transient ischemic attack.

**Table 5:** Comparison of studies in terms of dosing of antiplatelet drugs during aspirin treatment.

Study	Object	Population	Antiplatelet Drugs	Platelet Function Assessment Device/s	Time Points
Wiśniewski, Filipska, 2020 [27]	The prognostic value of high platelet reactivity in ischemic stroke depends on the etiology: A Pilot study	IS patients (*n* = 69)	ASA (150 mg/day)	Chrono-Log; Multiplate	24 h after the onset of stroke
Wiśniewski, Sikora, 2020 [28]	High on-treatment platelet reactivity affects the extent of ischemic lesions in stroke patients due to large-vessel disease	IS patients (*n* = 69); patients with large-vessel disease (*n* = 20); patients with small-vessel disease (*n* = 49)	ASA (150 mg/day)	Chrono-Log; Multiplate	24 h after the onset of stroke
Karepov, 2008 [29]	Plasma triglycerides as predictors of platelet responsiveness to aspirin in patients after first ischemic stroke	IS patients (*n* = 59)	ASA (dose range 75–325 mg/day)	PFA-100	3–26 months
Yi, Zhou, 2013 [30]	Aspirin resistance in Chinese stroke patients increased the rate of recurrent stroke and other vascular events	IS patients (*n* = 634)	ASA (at a dose of 200 mg/day for the first 14 days and then 100 mg/day)	Biodata-PAP-4	7–10 days after the initiation of acetylsalicylic acid Was 1–2 years
Yi, Han, 2017 [31]	Statin and aspirin pretreatment are associated with lower neurological deterioration and platelet activity in patients with acute ischemic stroke	IS patients (*n* = 1124)	ASA (200 mg/day for 14 days and 100 mg/day thereafter)	Flow cytometer	Measured on admission and during 7–10 days after admission Death during the first 3 months after admission
Tsai, 2011 [32]	Statin pre-treatment is associated with lowerplatelet activity and favorable outcome in patients with acute non-cardio-embolic ischemic stroke	AIS patients (*n* = 172); patients with pre-existing statin (*n* = 43); patients without pre-existing statin (cases with statins initiated post-stroke (*n* = 66) and without statin treatment (*n* = 63))	ASA (100 mg/day)	Flow cytometer	90 days after the acute stroke
Harrison, 2008 [33]	Lack of reproducibility of assessment of aspirin responsiveness by optical aggregometry and two platelet function tests	TIA or IS patients (*n* = 100)	ASA (dose range 75–150 mg/day)	Biodata-PAP-4; PFA-100 (CEPI); VerifyNow; LTA	Followed up 1 year after the presenting event
Sabra, 2016 [7]	Assessment of platelet function in patients with stroke using multiple electrode platelet aggregometry: a prospective observational study	IS patients (*n* = 70); Healthy (*n* = 72)	ASA (300 mg/day)	Multiplate	During treatment after admission to hospital and within 3–5 days of starting aspirin therapy
Kim, 2018 [34]	Clinical significance of acute and serial platelet function testing in acute ischemic stroke	AIS patients (*n* = 805)	ASA (300 mg/day and 100 mg/day after)	VerifyNow	After 3 h of ASA loading and on the fifth day of ASA administration
Jastrzębska, 2013 [35]	Factors influencing Multiplate whole blood Impedance Platelet Aggregometry measurements, during aspirin treatment in acute ischemic stroke: a pilot study	AIS patients (*n* = 133)	ASA (150 or 300 mg/day—first, all patients received 150 mg/day of ASA after 7 days, tests were performed and the dose of the drug was increased to 300 mg/day in refractory patients)	Multiplate (ASPItest)	7 days after starting treatment with ASA at a dose of 150 mg, then, if necessary, increase the dose to 300 mg and measured 7 days after starting treatment with the higher dose
Lee, 2010 [36]	Addition of cilostazol reduces biological aspirin resistance in aspirin users with ischemic stroke: a double-blind randomized clinical trial	IS patients (*n* = 192); citlostazol group (*n* = 90); placebo group (*n* = 102)	ASA (100 mg/day)	VerifyNow (ASA)	Before and after 4 weeks of therapy with ASA and cilostazol

AIS—acute ischemic stroke, ASA—aspirin, IS—ischemic stroke, LTA—light transmittance aggregometry, PAP-4D—optical platelet aggregometer, PFA—platelet function analyzer, TIA—transient ischemic attack.

**Table 6:** Comparison of studies in terms of dosing of antiplatelet drugs during clopidogrel treatment.

Study	Object	Population	Antiplatelet Drugs	Platelet Function Assessment Device/s	Time Points
Varvat, 2019 [37]	Monitoring of biological response to clopidogrel after treatment for non-cardioembolic ischemic stroke or transient ischemic attack	TIA patients (*n* = 72)	CLO (75 mg/day)	VASP; flow cytometer; LTA	For 24 h
Bagoly, 2013 [38]	Comparison of a new P2Y12 receptor specific platelet aggregation test with other laboratory methods in stroke patients on clopidogrel monotherapy	IS patients (*n* = 111); Control group (*n* = 140)	CLO (75 mg/day)	Chrono-Log; VASP; VerifyNow P2Y12	Within 1, 4 and 24 h
Qiu, 2015 [39]	Predictive value of high residual platelet reactivity by flow cytometry for outcomes of ischemic stroke patients on clopidogrel therapy	IS patients (*n* = 198)	CLO (75 mg/day)	VASP	Before and 7 days from the start of CLO therapy
Meves, 2014 [40]	Clopidogrel high-on-treatment platelet reactivity in acute ischemic stroke patients	AIS patients (*n* = 159); C-HTPR patients (*n* = 70)	CLO (75–300 mg/day); ASA (100–300 mg/day); ASA + CLO (not reported)	Multiplate	48 h after therapy
Rath CL, 2018 [41]	High on-treatment platelet reactivity in danish hyper-acute ischemic stroke patients	IS or TIA patients (*n* = 219)	CLO (300 mg/day)	VerifyNow P2Y12	8–24 h after CLO intake

AIS—acute ischemic stroke, ASA—aspirin, ASA + CLO—dual therapy, C-HTPR—clopidogrel high-on-treatment platelet reactivity, CLO—clopidogrel, IS—ischemic stroke, LTA—light transmittance aggregometry, TIA—transient ischemic attack, VASP—flow cytometer.

**Table 7:** Comparison of studies in terms of dosing of antiplatelet drugs.

Study	Object	Population	Antiplatelet Drugs	Platelet Function Assessment Device/s	Time Points
Coignion, 2015 [42]	Interest of antiplatelet drug testing after an acute ischemic stroke	AIS patients (*n* = 287); ASA patients (*n* = 156); CLO patients (*n* = 19); ASA + CLO (*n* = 112); APD sensitive group (*n* = 234); APD resistant group (*n* = 53)	ASA (not reported); CLO (not reported); ASA + CLO (not reported)	Multiplate	Not reported
Cha, 2016 [43]	High residual platelet reactivity (HRPR) for adenosine diphosphate (ADP) stimuli is a determinant factor for long-term outcomes in acute ischemic stroke with anti-platelet agents: The meaning of HRPR after ADP might be more prominent in large atherosclerotic infarction than other subtypes of AIS	AIS patients (*n* = 968); LAA patients (*n* = 442); SVO patients (*n* = 283); UD patients (*n* = 243)	ASA (300 mg/day); ASA + CLO (100 mg/day + 300 mg/day); ASA + CLO (75 mg/day + 100 mg/day)	OPA	5 days after hospital admission, 1-year follow-up

AIS—acute ischemic stroke, ASA—aspirin, ASA + CLO—dual therapy, CLO—clopidogrel, LAA—large artery atherosclerosis, OPA—optical platelet aggregometry, SVO—small vessel occlusion, UD—undetermined.

## Data Availability

Not applicable.
